# Preparation of a new coating of graphene oxide/nickel complex on a nickelized metal surface for direct immersion solid phase microextraction of some polycyclic aromatic hydrocarbons

**DOI:** 10.1186/s13065-021-00783-w

**Published:** 2021-10-16

**Authors:** Yalda Pasandideh, Habib Razmi

**Affiliations:** Department of Chemistry, Faculty of Basic Sciences, Azarbaijan Shahid Madani University, P.O. Box: 53714-161, Tabriz, Iran

**Keywords:** High-performance liquid chromatography, Solid-phase microextraction, Metal-based fiber, Nickel/graphene oxide, Nickel-complex coating, Polycyclic aromatic hydrocarbons

## Abstract

**Background:**

Solid-phase microextraction (SPME) is a versatile sampling and sample preparation technology that possess a significant application in the extraction and pre-concentration of a broad range of micro-pollutants from different kind of matrices. Selection and preparation of an appropriate fiber substrate and coating materials have always been the main challenges of the SPME procedure. This paper introduces a high-efficiency metal-based SPME fiber with a new chemical coating of nickel/graphene oxide/nickel tetraazamacrocyclic complex (Ni/GO/NiTAM).

**Result:**

The Ni/GO/NiTAM sorbent was electroless deposited onto the surface of an aluchrom (Alu) wire, and then the prepared fiber was employed for the extraction and pre-concentration of some PAHs before their HPLC–UV analysis. The prepared fiber characterization data were assessed using FE-SEM, EDX, XRD, FT-IR, and BET techniques. The method validation parameters, including the linearity range (LRs: 0.10 to 200.0 µg L^−1^), the limit of detection (LODs: 0.03‒0.30 µg L^−1^), and the limit of quantification (LOQs: 0.10–1.00 µg L^−1^), under optimal conditions. The relative standard deviations (RSDs) of intra-day, inter-day, and single fiber repeatability (for the samples spiked at 25 µg L^‒1^) were in the range of 0.32–2.94, 1.20–4.09, and 1.42‒4.39%, respectively. In addition, the technique recoveries (RR %) and enrichment factors (EF) were in the range of 83.10‒107.80% and 83–164, respectively.

**Conclusion:**

The fiber fabrication was simple, and the applied materials were also economical and easily accessible. Alu metal has high physicochemical and mechanical stability and thus can be a good alternative for the substrate of the fragile commercial SPME fibers. High rigidity and durability, long service life, and high extraction capability are some of the other advantages of the offered fiber.

**Supplementary Information:**

The online version contains supplementary material available at 10.1186/s13065-021-00783-w.

## Background

Polycyclic aromatic hydrocarbons (PAHs) are a big group of universal contaminants produced primarily during incomplete combustion of organic materials [1, 2]. PAHs contaminate the food chain from the air, soil, and water, or when food is prepared, preserved, stored, and cooked [3]. For example, vegetables and fruits, as one of the widely used food sources, might be infected with PAHs via proximity to highways and industrial areas or long-distance airborne transportation [4]. As another option, bread and cereals can be contaminated by PAHs through both raw materials (especially flour) and the baking process [5]. Bread baking with direct heat usually causes the rapid transfer of incomplete combustion contaminants to the dough and its final product [6]. In addition, food processing steps, including drying, grilling, roasting, smoking, and frying impressive in contamination of foodstuffs [7]. Since most PAHs are carcinogenic (especially bladder, esophageal, lung, and colon cancers) and mutagenic, there is a great deal of concern about them. Thus, their trace analysis and monitoring are of interest to researchers [8]. Due to the low levels of PAHs in environmental and food samples, doing a pre-concentration and extraction step(s) is usually essential before the main analysis to certify the accuracy and sensitivity of the desired method [9]. So far, various liquid-based and solid-based extraction techniques have been reported for the extraction and pre-concentration of PAHs from different samples [10–12]. Among them, the SPME technique has become more widely used in recent decades.

Solid phase microextraction (SPME) is a modern, fast, simple, and solvent-free sampling and sample preparation technology used for the extraction and pre-concentration of different organic micro-pollutants in food, environmental, biological, and pharmaceutical samples [13, 14]. SPME apparatus is generally made of a fused silica fiber (or rod) covered with a thin film of polymeric, solid, or liquid sorbents (such as polyacrylate (PA) and carboxene/polydimethylsiloxane (C/PDMS)) [15, 16]. Regardless of all the advantages of SPME, this technique has several fundamental drawbacks, including limited sorbents range, difficulty in finding an appropriate polarity coating, low thermal stability in GC analysis, swelling and stripping of coating (especially in organic solvents), fragility, bending of fiber substrates, low extraction performance and high prices [17]. Electrochemical and physical coating technologies, utilizing flexible metal substrates, and using new coating materials (such as carbon materials, aerogels, metal oxides, ionic liquids, and different pristine bio-materials) are some of the technical solutions that have been proposed to overcome these problems [18–20]. For example, numerous fibers based on the flexible metal wire substrates have been fabricated that can be employed with greater convenience because of their different physicochemical properties [21, 22]. Aluchrom metal alloy (Alu), as a new substrate for the SPME fiber, has just recently been introduced in our articles [23, 24]. In these works, Alu wires were applied once without any special pretreatment and coating material and once with a new coating of a pristine biomaterial and displayed high extraction and preconcentration efficiency in both cases. However, the coating of Alu wire with chemical materials and methods has not been studied yet. A sticky paste film (such as epoxy or nafion) is usually applied for the better attachment of coatings to the metal-based SPME substrates. On the other hand, many studies have been done to replace conventional coatings with new ones. For example, graphene oxide (GO) is a useful single atomic layered carbon compound that can be a potent substance for the adsorption and extraction methods due to its large specific surface area. Therefore, using GO in the pre-concentration, extraction, and microextraction procedures can efficiently improve their performance [25]. In addition, deposition of metal (M) or metal oxides (MO), such as three-dimensional transition metal oxides (3D-TMO), metal–organic frameworks (MOFs), and nano-crystalline mixed metal oxides (MMO), on the fiber substrate have developed many new SPME fibers [26, 27]. These structures demonstrate good adsorption and extraction characteristics and provide better adhesion between the fiber and its covering materials by creating active groups on the surface. Deposition processes are generally performed by electrochemical, electrophoretic, and anodizing techniques [28, 29].

Despite all these attempts, thermal instability, stripping of the coating materials, limited type of sorbents, and high costs of the commercial SPME fibers are not completely solved. Therefore, designing of a new, stable, non-fragile, and cost-effective SPME fiber with an easy fabrication process, high sensitivity, precision, and efficiency, and good adhesion of the coating to the fiber surface is still required.

In the present article, an electroless nickelized aluchrom surface coated with graphene oxide/nickel tetraazamacrocyclic complex (Alu-Ni/GO/NiTAM-SPME), as a novel concept in the fabrication of metal-based SPME fiber, was introduced. The suggested fiber efficiency has been successfully evaluated with high-performance liquid chromatographic (HPLC) analysis of some PAHs, as the model analytes, in different standard and real samples.

## Experimental

### Chemical and materials

Pure PAHs consist of naphthalene, acenaphthene, fluorene, phenanthrene, anthracene, fluoranthene, pyrene, and chrysene purchased from Merck (Darmstadt, Germany). A stock standard solution of PAHs has been prepared at a concentration of 100 mg L^–1^ in methanol. Acetonitrile and methanol with HPLC grade were from Carlo Erba (ValdeReuil, France). The HPLC-grade water and the other solvents and chemicals have been obtained from Merck (Darmstadt, Germany). The Alu wire was supplied from Simcat Co. in Tabriz (Iran) and employed as the SPME fiber substrate. Tomato, potato samples have been acquired from Seed and Plant Research Improvement Institute in Karaj (Iran). Sangak bread has been purchased from the Shahrenan-Jihot company (Karaj, Iran).

### Instruments and apparatus

A JASCO (Japan) HPLC system was used for the chromatographic experiments. The system was equipped with an isocratic PU-1580 pump, a UV-1575 ultraviolet detector (JASCO-1575), and a Rheodyne 7725i six-port switching valve (Rheodyne, Cotati, CA, USA). An HSS-2000 pack (JASCO) with an LC-Net II/ADC interface has controlled the chromatograph and a BORWIN software (version 1.50) processed all data. All the separation experiments have been achieved on an analytical ODS-3 column (250 mm × 4.6 mm ID, 5 μm) with an ODS-3 guard column (10 mm × 4 mm ID, 5 μm) (MZ-Analysentechnik, Germany). The injection of sample solutions into the HPLC system has been done with a 25 μL microsyringe (zero dead volume, Hamilton, Switzerland). A Tescan mira3 electron microscope (Brno-Czech Republic), an FT-IR system (Bruker, Ettlingen, Germany) spectrometer, a Bruker D8 Advance X-ray apparatus (Bruker AXS, Karlsruhe, Germany), and a Brunauer-Emmet-Teller (BET) technique with a Gemini 2375 micrometric instrument and N_2_ adsorption–desorption analysis were employed for the characterization studies. Moreover, an ultrasonic device (Falc instrument S.r.I, LBS2, Italy), a Metrohm pH meter model 744 (Switzerland), a Beckman GS-6 centrifuge (USA), an IKA RCT basic magnetic stirrer (Germany), and an oven were employed. STARCKE P220 and P400 waterproof sandpapers have been purchased from a local store in Tabriz (Iran). All the analysis steps have been performed at laboratory temperature. The chromatographic data have been accomplished under isocratic conditions. The wavelength of the UV detector was set at 254 nm.

### Fabrication of the Alu-Ni/GO/NiTAM-SPME fiber

The Alu wires were polished with P220 and P400 sandpapers, respectively, to remove unpleasant imperfections from the surface, and a relatively mirror-like finish was achieved. Then it was washed with deionized water and sonicated in a mixture of acetone: water (1:1) and dried in air. Then a chemically etching step was performed by immersing 2 cm of the polished surface in 1 M NaOH for 1 min. In the next step, the polished and etched wires were coated by the Ni/GO/NiTAM sorbent. For this purpose, at first, the electroless deposition of metallic nickel on the fiber surface was accomplished by dipping one polished and etched Alu wire into the plating solution containing 1 M NiCl_2_ and 2 M NH_4_Cl at pH = 1, for 5 min [30]. The fiber had been taken out and rinsed with deionized water. Then, the nickel-coated fiber was immersed directly in a dispersion of GO in HPLC-grade water (containing 10% N-methyl-2-pyrrolidone and 10% ethylene glycol) for 45 min, under continual agitation (600 rpm). GO was synthesized by the conventional Hummers’ method [31]. The obtained Ni/GO-covered Alu-fiber (Alu-Ni/GO-SPME) was pulled out and dried in the air. In the next step, the Alu-Ni/GO-SPME fiber has been soaked in a solution of Ni-tetraazamacrocyclic complex (3 mg mL^−1^) at pH = 12 for 30 min (T = 70 °C) [32]. Finally, the fiber was removed, washed with deionized water, and dried at 30 °C to fix a suitable thickness of the Ni/GO/NiTAM coating on its surface.

### Microextraction procedure

25 mL aqueous standard/sample solution containing the selected PAHs (at a concentration of 25 μg L^−1^) were transferred into a sample container, placed on a magnetic stirrer, and agitated with a magnetic bar. The fabricated Alu-Ni/GO/NiTAM-SPME fiber was directly dipped into the solution. The sample was extracted through the direct immersion mode (DI), and adsorption equilibrium was achieved after 30 min, under frequently stirring (800 rpm). Then the fiber was withdrawn and immediately placed in 1 mL acetone for 10 min to desorb the analytes. Next, the SPME fiber has been pulled out from acetone, and the residual compounds were dried by N_2_ gas. Drying the sample was done to prevent dilution effects, improve the technique sensitivity, and compatible the desorption solvent with HPLC mobile phase. The dried extract was then dissolved in 100 μL acetonitrile and injected into the HPLC system for the final analysis. The isocratic elution of analytes was performed utilizing a mixture of acetonitrile: HPLC-grade water (75:25, v/v) at the flow rate of 1 mL min^−1^.

### Preparation of real samples

In this work, tomato, potato, and Sangak Persian bread were chosen as the real samples. The tomato sample was washed nicely to remove all dirt and unwanted substances from the outer layer and then cut into two pieces. Then a half piece of the sample has been peeled and grated very finely, and the other half grated with the peel. The potato sample has been prepared the same as the tomato sample. Sangak is traditional Iranian bread that is baked by the direct (on a bed of small river stones in a tandoor or oven) and indirect (in industrial bread machines) heating process. The heating method, fuel type, and temperature affect the amount of bread contamination (especially PAHs). Thus, two available types of this bread with similar ingredients have been purchased. So far, various methods have been reported for the preparation of vegetables and bread samples during PAHs analysis [33–37]. Briefly, 5 g of each of these six samples was ultrasonicated twice with 2 mL of an appropriate solvent for 10 min. A mixture of acidified acetonitrile (with 1% acetic acid) and n-hexane: dichloromethane (1:1, v/v) solvents were chosen for the extraction of PAHs from the vegetables and bread samples, respectively. In all cases, the attained mixtures were centrifuged for 15 min at 4000 rpm, and then the upper solutions were overflowed, mixed up, and dried by nitrogen gas. Finally, the dried extracts were dissolved in 1 mL methanol and diluted with DDW to a final volume of 25 mL. The obtained solutions have been used for the DI-SPME-HPLC analysis of PAHs.

### Method validation

Method validation is an essential process that demonstrates an analytical procedure is suitable for the desired purpose in terms of quality, reliability, and consistency of results. At the present study, ICH Q2 (R1) was applied for the validation [38], including 10 different concentrations for the linearity, 10 determinations covering the specified range for the procedure (2 concentrations/5 replicates each) for precisions, five repeated analysis of blank samples for limit of detection (LOD) and limit of quantification (LOQ) and spike the real samples with known quantities of the target analytes for the accuracy and recovery studies. Based on the requirements of ICH Q2 (R1), the robustness of the method was also considered under study. In addition, Mandel’s fitting test has been done to investigate the linearity of the calibration curves. Mandel’s fitting test is suggested by IUPAC and calculates from the difference of the variance of the residual standard deviation of linear (S_y1_) and the potential second-order (S_y2_) calibration models. This is compared with the standard deviation of the potential second-order calibration model using the F-test (Eq. (1)).

1 $$ F_{experimental} = \frac{{S_{y1 }^{2} \times \left( {n - 2} \right) \times S_{y2}^{2} \times \left( {n - 3} \right)}}{{S_{y2}^{2} }} $$

## Results and discussion

### Characterization of the Alu-Ni/GO/NiTAM-SPME fiber

**Fig. 1:**
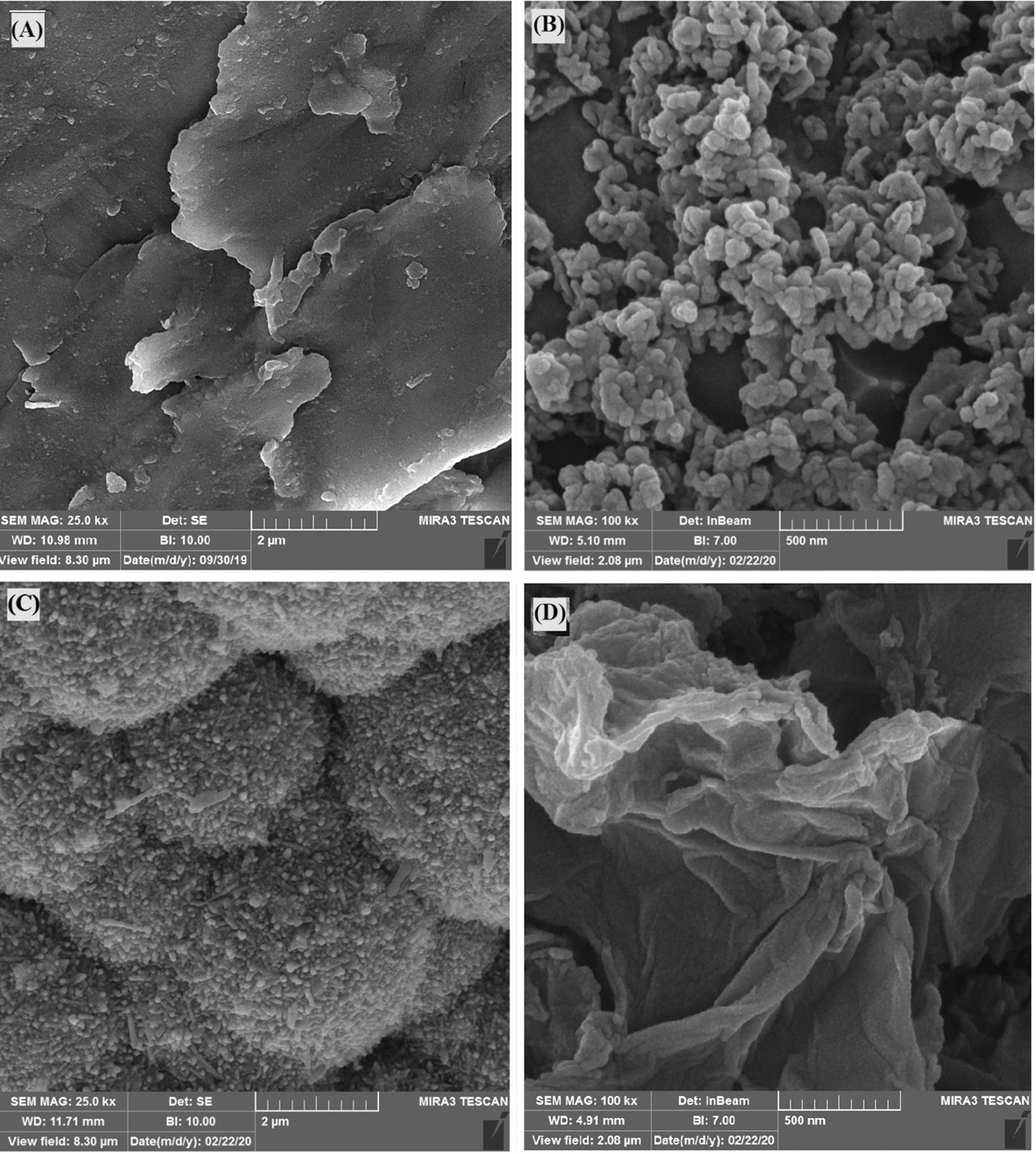
FE-SEM micrographs of the **A** Alu-SPME (×50,000), **B** chemically etched Alu-SPME (×100,000), **C** Alu-Ni-SPME (×25,000) and **D** Alu-Ni/GO/NiTAM-SPME (×25,000) fibers

The prepared Alu-Ni/GO/NiTAM-SPME fiber was characterized utilizing FE-SEM, EDX, XRD, FT-IR, and BET techniques. The FE-SEM spectroscopy was applied to investigate the surface morphology and pores structure of the polished Alu-SPME fiber without any coating, chemically etched Alu-SPME, Alu-Ni-SPME, and Alu-Ni/GO/NiTAM-SPME fibers. As can be seen in Fig. 1A, the polished Alu-SPME fiber, without any special pretreatment and coating, has a relatively smooth structure. However, the chemical etching has created branch-like patterns and irregular pores on the fiber surface (Fig. 1B). Such structure is resulted in creating relative adsorption properties and, more importantly, improvement of the adhesion of sorbent to the fiber surface. In addition, the micro-image of Alu-Ni-SPME fiber (Fig. 1C) illustrates a surface with an attractive collection of abundant and uniformly porosities, which can significantly increase the fiber adsorption attributes. Finally, the positive effect of the GO/Ni-complex on the sorbent porosities enhancement is proved in Fig. 1D. According to this micro-image, the GO groups alongside the Ni-complexes have created folds, crinkles, and rolled edges [32] that amplified the adsorption properties. The thicknesses of Ni, Ni/GO, and Ni/GO/NiTAM layers were 12, 48, and 92 µm, respectively.

**Fig. 2:**
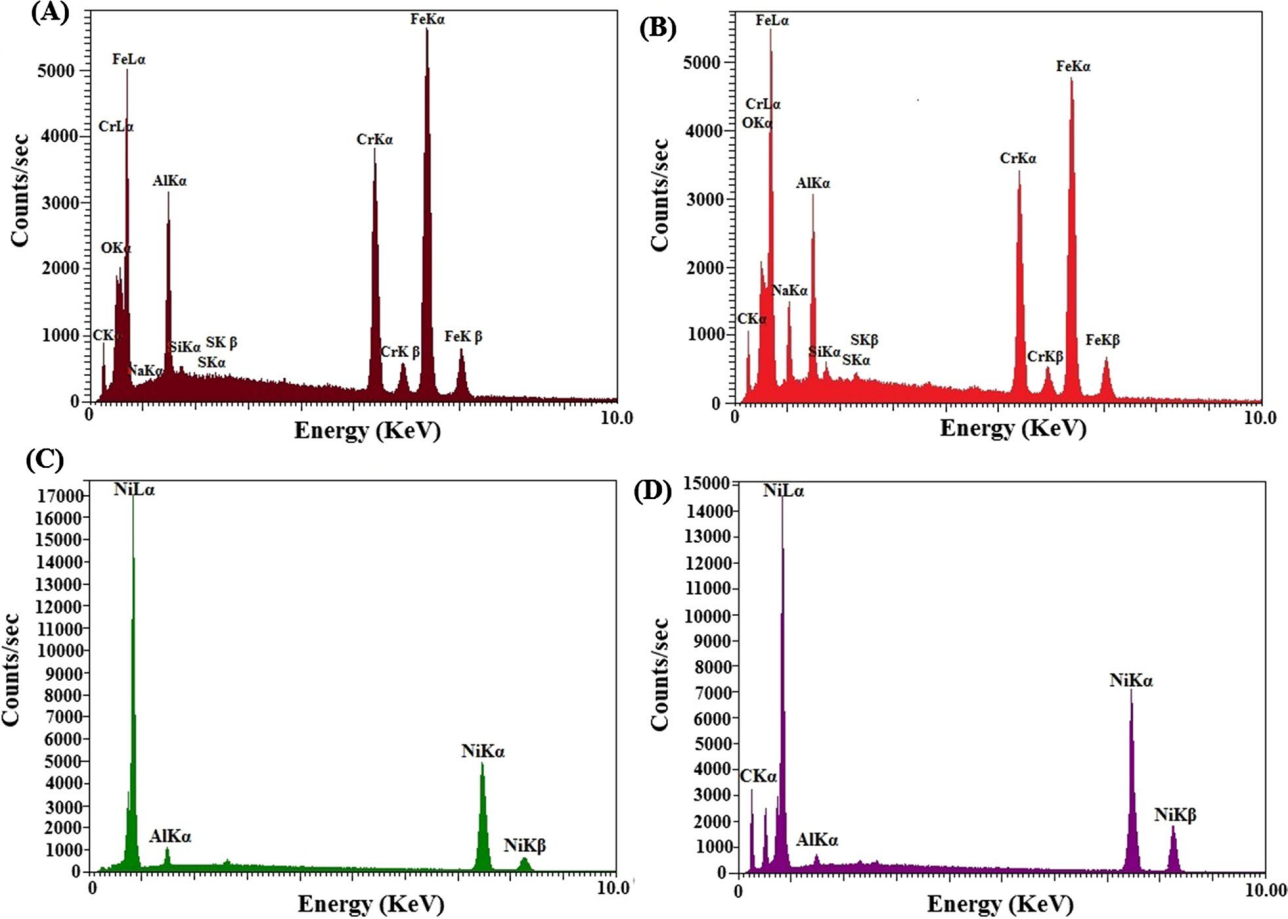
EDX patterns of the prepared **A** Alu-SPME, **B** chemically etched Alu-SPME, **C** Alu-Ni-SPME and **D** Alu-Ni/GO-SPME fibers

In addition, chemical micro-analysis and elemental characterization were recorded employing EDX spectroscopy for the polished Alu-SPME, chemically etched Alu-SPME, Alu-Ni-SPME, and Alu-Ni/GO/NiTAM-SPME fibers. The main peaks of Alu elements are visible in the uncoated-Alu-fiber spectrum (Fig. 2A). The next pattern is related to the chemically etched Alu-fiber (Fig. 2B). The slight increase in the Alu-peaks intensity is probably due to the etching process, which has allowed access to the bottom parts of the substrate by removing the surface layers or possible surface contaminants. The appearance of sharp and high-intensity peak of nickel and the reduction of the Alu-peaks intensity in Fig. 2C indicate that the fiber surface is well-coated with the Ni-plating solution. Finally, concerning Fig. 2D, the carbon peak of GO is visible accompanied by increasing the intensity of Ni-peaks, which proves the proper covering of fibers with the coating materials.

**Fig. 3:**
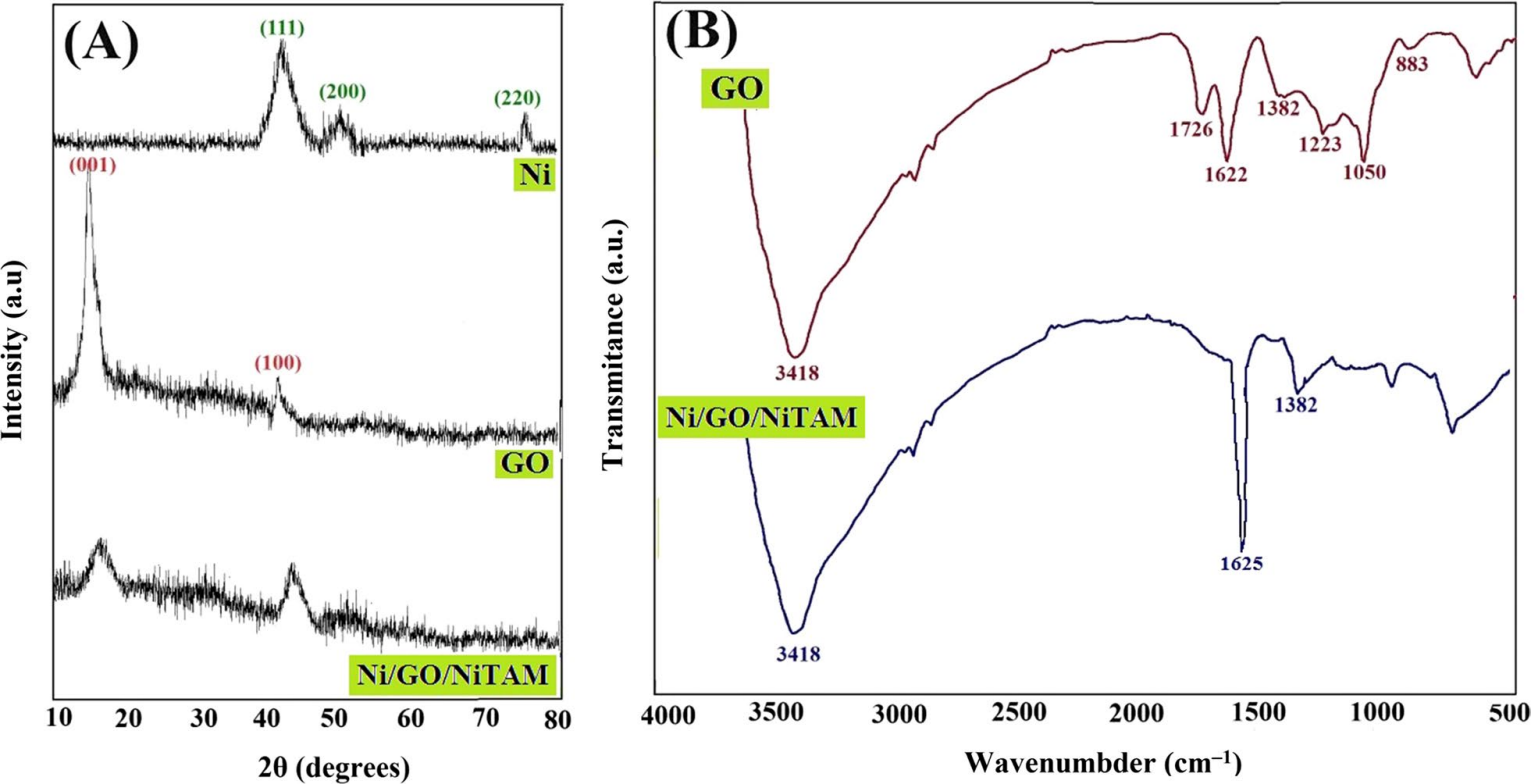
EDX patterns of Ni, GO and Ni/GO/NiTAM coating (**A**) and FT-IR spectra of GO and Ni/GO/NiTAM coating (**B**)

The XRD patterns of the neat Ni, synthesized GO and Ni/GO/NiTAM coating are shown in Fig. 3A. For Ni, the characteristic peaks observed at around 44°, 52°, and 76° are ascribed to the (111), (200), and (220) atomic structures of Ni, respectively [39]. For GO, the diffraction peaks at around 2θ = 11.8° and 43° have corresponded to the (001) and (100) crystallographic planes of GO [34]. The interlayer spacing of GO sheets was about 0.78 nm, which has been created by the oxygen-containing groups and trapped water molecules [40]. For Ni/GO/NiTAM, decreasing the GO and Ni peaks intensities is related to the incomplete reaction of GO and Ni groups during the coating process. Therefore, a strong linkage has been created between the abundant oxygen-rich functional groups of GO and Ni nucleating parts.

The FT-IR spectra of the GO and Ni/GO/NiTAM are also demonstrated in Fig. 3B. The stretching vibration band of hydroxyl, related to the CO–OH and C–OH groups, was appeared at 3418 cm^‒1^. The peaks at 1726 and 1622 cm^‒1^ are corresponded to the C=O and C=C stretching vibrations, respectively. The stretching vibration bands of C–O also appeared at 1382, 1223, 1050, and 883 cm^‒1^. In the case of Ni/GO/NiTAM, the sharp absorption band of carboxylate ions related to the GO-Ni-complex has appeared at 1625 cm^−1^. In addition, the absorption band of 1382 cm^‒1^ was become more intense, which corroborates the well-known complexation interaction between the metal and GO [41]. Such interaction between the Ni and GO groups has been led to the deposition of GO on the presented SPME fiber surface.

The BET analysis of the proposed fiber has been also done. The specific surface area, total pore volume, and mean pore diameter of the Ni coating were 11.355 m^2^ g^–1^, 0.068 cm3 g^–1^, and 18.912 nm, respectively. However, addition of GO onto the Ni surface has changed these values to 36.801 m^2^ g^–1^, 0.339 cm3 g^–1^, and 9.751 nm, respectively. The results demonstrate that the surface area and pore volume of the Ni/GO/NiTAM coating is increased significantly by the incorporation of the GO nanoparticles, while the pore diameter decreased. Increasing the Ni/GO/NiTAM coating surface area is probably related to the higher pore diameter of the GO compared to the Ni. Therefore, by increasing the surface area and decreasing the pore size, it is expected that the Ni/GO/NiTAM coating possesses a higher extraction capability compared to the GO.

### Optimization of the proposed method

The present study has been included two separate stages: (a) the fabrication of an SPME fiber with new coating materials and (b) utilizing the achieved fiber in the analysis of some PAHs. Therefore, optimizations of both of these stages were necessary to access a high-performance microextraction technique.

For this purpose, initially, the best conditions for the fabrication of a high-quality Alu-fiber have been determined. In this step, the other analysis factors were constant at sample volume: 25 mL; sample concentration: 25 µg L^‒1^; extraction time: 60 min; desorption solvent: acetonitrile; desorption solvent volume: 2 mL, desorption time: 30 min, and stirring rate: 200 rpm. After the fabrication of a suitable fiber (Alu-Ni/GO/NiTAM-SPME), the significant experimental parameters have been optimized. The optimized factor in each step was applied in the further stages. Additional file 1: Figures S1 and Additional file 2: Figure S2 display the gradual improvement of the chromatographic signal intensity and the increase of method performance by the optimization of the effective parameters.

#### Optimization of the important factors affecting the fiber fabrication quality

In this part, some important factors influencing the fiber efficiency, coating thickness, and coating adhesion have been optimized to get a high-quality fiber. In this regard, different affecting factors were optimized, such as the kind of coating, etching reagent, etching time, concentration of NiCl_2_, pH of plating solution, time of dipping fiber into the plating solution, GO dispersion, pH of GO dispersion, stirring rate of dispersion and time of immersing fiber into the GO dispersion.

The kind and properties of the coating are key factors affecting the sensitivity, selectivity, and reproducibility of the SPME efficiency [22]. For evaluating this parameter, four similar Alu-wires were polished with sandpapers and coated with different coating materials. One of these wires was Ni-covered by immersing the polished surface into the plating solution (1 M NiCl_2_ + 2 M NH_4_Cl). The second wire was GO-covered by dipping the fiber into the GO dispersion. The third wire was first immersed into the plating solution. Then the fiber was withdrawn and dipped into the GO dispersion to the final shape of fiber (Ni/GO-covered) was made. The last wire has been prepared like the previous fiber, and then the Ni/GO-covered-fiber was coated by soaking in the Ni-complex solution. Eventually, the performance of these fibers has been studied for the extraction, pre-concentration, and determination of target analytes. The results showed that Ni/GO-SPME fiber possesses good absorption properties, but adding a new layer of Ni-complex to the surface, significantly increases its adsorption properties (Additional file 1: Fig. S1A).

Etching is the cutting process of a metal surface by a strong acid or base or mordant to create a special design [42]. Xu and his co-workers have introduced the first etched-SPME fiber in 2009 and applied it for the extraction and pre-concentration of some PAHs from water samples [43]. In the microextraction procedures, etching of the metal substrate increases the specific surface area by creating roughness and porosity. Accordingly, the adhesion between the fiber and its coating enhance, and the technique performance improves. In the present study, different reagents such as HF, HCl, H_2_SO_4_, and NaOH at a concentration of 1 M have been tested to find a suitable etching agent. As the results show (Additional file 1: Fig. S1B), the best signal intensity was obtained by NaOH. The kind of substrate and the solvent strength determine the required time for the etching process. Generally, the etching procedures are involved with two basic challenges: incomplete-etching and over-etching. In incomplete-etching, time is not adequate for the complete removal of materials, and thus the desired porosities and roughness not possibly create. Against, too intensive etching (over-etching), particularly too long-time etching process usually damages the surface of the substrate and reduces the adhesion [44]. Each of these challenges reduces the method efficiency. Here, the effect of etching time has been examined in the range of 0.5‒5 min. Based on the outcomes (Additional file 1: Fig. S1C), the etching time of 1 min has been selected for further studies.

Besides, the thickness of the fiber coating affects the adsorption capacity and extraction efficiency. The thicker coating offers more extraction capability through its greater specific surface area. However, the thinner coating offers a faster mass transfer of analytes [45]. The thickness of the covering film mainly depends on the concentration of coating materials and the dipping time of fiber in the coating solution. Therefore, optimization of covering conditions is essential. As mentioned, Ni/GO/NiTAM was the most suitable fiber coating. Since three steps were required to achieve such coating, optimization of all of these steps was essential (Additional file 1: Fig. S1D‒1SJ). So at first, the conditions of the plating solution, such as the concentration of NiCl_2_, pH, and the time of electroless Ni-coating, have been optimized. Then the concentration of the GO dispersion, pH, stirring rate, and GO coating time has been optimized. The addition of NMP and ethylene glycol to the GO dispersion increases its stability and provides better coating conditions in a shorter time [46]. Coating conditions of the fiber with Ni-complex have been done according to Basiuk’s report [32]. By the results, the optimum conditions for coating the Alu-SPME fibers were as follows:

(I) Plating solution: concentration: 2 M, pH: 1, time: 10 min.

(II) GO dispersion: concentration: 10 mg mL^‒1^, pH: 7, stirring rate: 600 rpm and time: 50 min.

(III) Ni-tetraazamacrocyclic solution: concentrating: 3 mg mL^‒1^, pH: 12, time: 30 min, T: 70 °C.

#### Optimization of the significant experimental factors

After the fabrication of a suitable fiber, the effect of the important analyzing factors have been studied. The most significant parameters optimized in this work were the extraction time, desorption solvent, volume of desorption solvent, desorption time, pH of sample solution, stirring rate, and ionic strength.

Since the SPME is an equilibrium-based technique, the extraction time is a significant factor that affects the extraction yield. The optimal extraction time is the shortest duration needs to adsorb the maximum amount of analytes onto the fiber surface [47]. At the present experiment, the influence of the extraction time has been inspected in the range from 10 to 60 min. According to Additional file 2: Fig. S2A, the extraction efficiency has been increased as the extraction time increased up to 30 min, and then it was remained unchanged. Therefore, 30 min was selected as the suitable extraction time.

The complete desorption of analytes will increase the sensitivity of the microextraction procedures. Hence, the selected desorption solvent should possess a high ability to desorb the analytes completely or as much as possible. Acetonitrile, acetone, methanol, THF, and n-hexane have been tested to find the appropriate desorption solvent. According to the results (Additional file 2: Fig. S2B), acetone was displayed the highest desorption potential and therefore selected for this step.

The large volume of the desorption solvent is problematic for the environment and additionally not economical. Versus, the low solvent consumption may cause the incomplete desorption of analytes. Therefore, the volume of desorption solvent ought to be optimized to determine the lowest amount of solvent with the maximum desorption recovery. The effect of desorption solvent volume on the method recovery has been investigated in the range of 0.5‒2.0 mL. As can be seen in Additional file 2: Fig. S2C, the recoveries have been increased significantly from 0.5 to 1.0 mL but not notably different between 1.0 and 2.0 mL. Hence, the optimal volume of 1 mL has been chosen for the desorption step.

In addition, desorption time has been studied to determine the minimum duration required for the complete desorption of analytes from the sorbent. This factor was studied in different desorption times varied from 5 to 20 min. The results (Additional file 2: Fig. S2D) demonstrated that 10 min was the proper desorption time for all the target analytes.

pH of sample solutions is one of the other factors that affect the extraction and adsorption procedures. Theoretically, the sample pH can increase the extraction proficiency of acidic and basic analytes by decreasing their solubility [48]. In addition, for the metallic SPME fibers, sample pH can influence the chemical stability, surface structure, and surface charges of the fibers substrate and coating. Here, pH variations have not affected the PAHs ionization but they could potentially affect the fiber substrate and its coating. Therefore, optimization of this parameter was necessary. The effect of pH on the extraction yield has been evaluated at different pH values from 3 to 11. Based on the results (Additional file 2: Fig. S2E), all of the experiments were performed at pH = 7. Adjustment of pH was performed by adding appropriate amounts of diluted HCl and NaOH to the sample solutions.

In general, the diffusion layer around the SPME fiber reduces at a higher stirring rate, and consequently, the mass transfer of analytes enhances [49]. In this work, the effect of stirring rate on the technical efficiency has studied in the range from 0 to 1000 rpm. The positive influence of the stirring rate on the chromatographic signals has been obtained at 800 rpm (Additional file 2: Fig. S2F). Thus, 800 rpm was set as the optimized stirring rate for more analysis.

### Memory effect

The memory (carry-over) effect is not a serious problem in the equilibrium-based microextraction techniques. However, on-fiber carry-over can be the main concern for the samples containing low concentration of desired analytes [50]. To assess this parameter, a second desorption step has been consecutively done after the primary adsorption. With the analysis of the related chromatograms, no noticeable carry-over was observed (Additional file 3: Fig. S3). These results prove that all the analytes have been completely removed in the first desorption step.

### Results of the method validation examination

The validation data of the developed SPME method have been evaluated at the optimum conditions, and the results were summarized in Table 1. The wide linear ranges (LRs) from 0.10 to 200.00 µg L^−1^ with good linearity (R^2^ > 0.99) have been achieved for the entire target PAHs. The slope of the calibration curves and their uncertainty are also presented in this table. For all the target analytes, the F_experimental_ (for the confidence level of P = 95%) was extracted from the experimental calibration and proved the linearity of the calibration curves. The LOD and LOQ of the technique were in the range of 0.03–0.30 and 0.10–1.00 µg L^−1^, respectively. The relative standard deviation (RSDs) of intra-day and inter-day for five repeated real samples spiked at two different concentration levels (1 and 25 μg L^–1^) were in the ranges of 1.42‒4.82% and 3.75‒7.29% (for the concentration of 1 µg L^−1^) and 0.32–2.94% and 1.20–4.09% (for the concentration of 25 µg L^−1^), respectively. The repeatability of the estimated single fiber represented RSDs value in the range of 3.58‒8.70 (for the concentration of 1 µg L^−1^) and 1.42‒4.39% (for the concentration of 25 µg L^−1^). Moreover, five new fibers were prepared on the same day, under the same conditions, and stored at laboratory ambient. In this case, the adsorption efficiency of fibers has been examined after 1 month. The results did not illustrate any reduction in the fiber performance, and the RSDs values were in the range of 0.39‒5.30% and 0.17‒2.93% for the spiked samples at the concentration of 1 and 25 µg L^−1^, respectively. The enrichment factor (EF), based on the ratio between the analyte concentration in the extraction phase and its primary concentration, was in the range of 83–164. The repeatability of the Alu-Ni/GO/NiTAM-SPME fiber was perfect when a first-hand Alu wire was employed. The fiber fabrication procedure was easy, and all the fibers were prepared manually. By fully following the preparation points and steps, the fabricated fibers were very similar and over 95% of the products offered nice repeatability. It should mention that the physical polishing of the sorption layer from the fiber surface and re-coating the second-hand Alu wires create poor repeatability. In this condition, about 35% of fibers are faulty, and thus the fibers with similar properties should be selected to provide acceptable repeatability. Furthermore, a reusability examination of the single fiber illustrated that, up to 25 repetitive measurements, there were no meaningful changes in the recovery results. However, after that, about 6.3% of fiber's efficiency has been diminished. However, in this study, each fiber was not employed more than 10 times to attain good recoveries and repeatable results.

**Table 1:** Analytical figures of merit of the developed method utilizing the Alu-Ni/GO/NiTAM-SPME fiber (n = 5)

Compounds	Analytical figures													
	LR^a^ (µg L^–1^)	Slope ± Un^b^	R^2c^	LOD^d^ (µg L^–1^)	LOQ^e^ (µg L^–1^)	RSD^f^ (%) (at the concentration of 1 µg L^–1^)				RSD (%) (at the concentration of 25 µg L^–1^)				EF^g^ ± RSD
						Intra-day	Inter-day	After 1 month	Single fiber	Intra-day	Inter-day	After 1 month	Single fiber	
Naphthalene	0.50‒25.00	1218.60 ± 0.15	0.9913	0.15	0.50	2.21	3.75	0.51	3.58	0.32	1.80	0.17	2.21	83 ± 3
Acenapthene	0.50‒100.00	1495.20 ± 0.11	0.9938	0.05	0.16	2.44	5.95	0.39	5.51	0.53	3.10	0.26	3.30	124 ± 2
Fluorene	0.10‒200.00	2813.50 ± 0.09	0.9925	0.20	0.66	3.00	6.12	3.06	8.70	1.18	4.05	2.40	4.39	97 ± 7
Phenanthrene	0.10‒150.00	2277.90 ± 0.08	0.9933	0.03	0.10	4.82	5.91	4.13	6.05	1.96	2.38	1.62	3.05	104 ± 5
Anthracene	0.10‒200.00	4724.90 ± 0.05	0.9915	0.03	0.10	2.61	4.00	1.59	4.83	0.84	1.20	0.33	2.78	94 ± 2
Fluranthene	0.50‒200.00	1537.60 ± 0.09	0.9919	0.15	0.50	3.20	4.38	3.04	4.22	1.31	2.87	1.16	1.42	118 ± 3
Pyrene	1.00‒200.00	1812.60 ± 0.01	0.9912	0.30	1.00	4.35	7.29	2.98	6.48	2.94	4.09	2.13	3.90	164 ± 7
Chrysene	0.50‒150.00	2069.30 ± 0.06	0.9920	0.07	0.23	1.42	6.11	5.30	7.49	0.98	3.62	2.93	3.71	135 ± 4

^a^Linear range

^b^Uncertainity

^c^Square of correlation coefficient

^d^Limit of detection

^e^Limit of quantification

^f^Relative standard deviation

^g^Enrichment factor

### Real sample analysis

The Alu-Ni/GO/NiTAM-SPME fiber was utilized for the DI-SPME-HPLC analysis of some PAHs in tomato, potato and, Sangak breed samples. Initially, the prepared samples were evaluated for the presence or absence of the aim analytes. Then, the samples were spiked with the standard solution of PAHs in two different levels of 5.00 and 10.00 µg L^–1^. Finally, the simultaneous analysis of the prepared samples has been performed in five replicated measurements (n = 5). The analytical outcomes for the quantitative determination of target PAHs in the real samples and their uncertainty (based on RSD %) have been reported in Table 2. The uncertainty of the obtained recoveries is equal to the RSDs calculated from the n-fold (n = 5) investigation in this step. The results proved that the outer parts of the vegetables and fruits are more contaminated, and the amount of PAHs in the unpeeled samples was more than peeled samples. In the case of bread samples, the bread baked in the tandoor (direct heat) has been more polluted than those baked in the bread machine (indirect heat). Given that good recoveries were achieved in all cases (82.90‒107.80%), it can conclude that the proposed method has acceptable performance in the simultaneous measurement of PAHs in different media and can be employed for the routine analysis of these compounds.

**Table 2:** The relative recoveries of the target PAHs determination utilizing the developed method (n = 5)

Analytes		Naphthalene			Acenapthene			Fluorene			Phenanthrene			Anthracene			Fluoranthene			Pyrene			Chrysene		
Samples	Added (µgL^–1^)	0.00	5.00	10.00	0.00	5.00	10.00	0.00	5.00	10.00	0.00	5.00	10.00	0.00	5.00	10.00	0.00	5.00	10.00	0.00	5.00	10.00	0.00	5.00	10.00
Peeled tomato	Found (µgL^–1^)	8.32	12.48	18.13	6.08	10.93	15.20	‒	4.27	9.72	8.91	13.21	18.52	‒	5.28	8.31	‒	4.2	8.63	‒	4.97	10.14	‒	4.83	9.79
	RR^a^ (%) + RSD (%)	‒	83.20 ± 2	98.08 ± 1	‒	97.00 ± 2	91.20 ± 2	‒	85.40 ± 3	97.20 ± 1	‒	86.50 ± 3	96.10 ± 3	‒	105.6	83.10	‒	84.00 ± 2	86.30 ± 3	‒	99.40 ± 3	101.40 ± 1	‒	96.60 ± 1	97.90 ± 2
Unpeeled tomato	Found (µgL^–1^)	13.3	18.13	22.14	‒	4.17	9.49	‒	5.05	9.36	14.06	19.00	24.20	‒	4.61	9.16	3.28	8.52	12.20	‒	5.03	10.55	‒	8.56	9.61
	RR (%) + RSD (%)	‒	96.60 ± 3	88.40 ± 2	‒	83.40 ± 3	94.90 ± 2		101.00	93.60	‒	98.80 ± 2	101.40 ± 1	‒	92.20	91.60	‒	104.80 ± 1	89.20 ± 2	‒	100.60 ± 1	105.50 ± 3	‒	85.60 ± 4	96.10 ± 2
Peeled potato	Found (µgL^–1^)	‒	4.46	10.37	5.30	10.43	15.41	‒	8.99	89.90	‒	4.16	10.12	‒	4.89	9.46	‒	4.75	8.61	‒	5.14	9.96	2.84	7.20	12.14
	RR (%) + RSD (%)	‒	89.20 ± 1	103.70 ± 1	‒	102.60 ± 1	101.10 ± 3	‒	90.11 ± 4	91.10 ± 2	‒	83.20 ± 2	101.20 ± 3	‒	97.80	94.60	‒	95.00 ± 2	86.10 ± 2	‒	102.80 ± 2	99.60 ± 3	‒	87.20 ± 3	93.00 ± 3
Unpeeled potato	Found (µgL^–1^)	‒	4.52	9.33	4.28	9.20	13.80	‒	10.35	8.79	8.24	12.62	17.68	‒	4.36	9.96	‒	4.63	9.02	‒	4.74	8.35	‒	8.29	9.44
	RR (%) + RSD (%)	‒	90.40 ± 3	93.30 ± 2	‒	98.40 ± 3	95.20 ± 1	‒	103.50 ± 3	87.90 ± 2	‒	87.60 ± 3	94.00 ± 3	‒	87.20	99.60	‒	92.60	90.20	-	94.80 ± 2	83.50 ± 4	‒	82.90 ± 3	94.40 ± 2
Machine Sangak	Found (µgL^–1^)	‒	4.45	10.44	‒	4.17	9.20	‒	4.90	8.88	3.75	8.25	12.69	‒	4.44	10.08	‒	4.30	8.62	‒	4.63	8.90	‒	5.03	9.09
	RR (%) + RSD (%)	‒	89.00 ± 2	104.40 ± 1	‒	83.40 ± 2	92.00 ± 3	‒	98.00 ± 1	88.80 ± 4	‒	90.00 ± 2	89.40 ± 1	‒	88.80	100.8	‒	86.00 ± 3	86.20 ± 2	‒	92.60 ± 3	89.00 ± 4	‒	100.06 ± 1	90.90 ± 2
Tandoori Sangak	Found (µgL^–1^)	‒	4.23	9.80	5.30	10.20	14.75	‒	4.00	8.47	10.37	15.13	19.02	9.18	14.57	18.22	‒	4.98	8.58	2.80	7.95	12.82	‒	5.00	9.19
	RR (%) + RSD (%)	‒	84.60 ± 1	98.00 ± 3	‒	98.00 ± 3	94.50 ± 1	‒	80.00 ± 4	84.70 ± 3	‒	95.20 ± 2	86.50 ± 3	‒	107.8	90.40	‒	99.60 ± 1	85.80 ± 2	‒	103.00 ± 2	100.20 ± 1	‒	100.00 ± 1	91.90 ± 3

^a^Relative recovery

‒: ND

Besides, the chromatograms obtained by the Alu-Ni/GO/NiTAM fiber for the tandoori Sangak samples, before and after spiking with 25 µg L^‒1^, of the target PAHs are shown in Additional file 4: Fig. S4. As can be seen, the bread sample was contaminated with acenaphthene, phenanthrene, anthracene, and pyrene that are most likely related to the incomplete combustion products.

Given all the obtained results, the good extraction capacity of the fiber is probably due to the surface high porosities, high surface area, weak π–π interaction between the C-bonds of PAHs and GO surface, H-π interaction between the ‒OH or ‒COOH groups of GO (hydrogen donor) and PAHs (hydrogen bond acceptor), and the interaction between the oxygen-containing functional groups of GO (n-electron donor) and the analytes (π-electron acceptor) [24]. The interaction between the nitrogen groups of the Ni-complex with PAHs can also create adsorption prpoperties. In addition, coordination interaction between Ni-atoms and Ni-ligands in Ni/GO/Ni-complex with PAH molecules is probably another factor influencing the adsorption and extraction efficiency of the fiber [51].

### Robustness of the proposed method

At first, the robustness of the Alu-wire has been examined at different conditions. For this purpose, a number of new Alu-wires were separately stored at various temperatures in the range of 20 to 200 °C for 2 h to investigate their thermal stability. In addition, the fiber chemical stability has been studied by individually immersing a few new Alu-wires in acetone, acetonitrile, deionized water, methanol, n-hexane and THF for 6 h (at laboratory temperature = about 30 °C). Then all of these fibers were coated by Ni/GO/NiTAM sorbent and used for SPME-HPLC analysis of target PAHs. No significant changes were observed in the offered method efficiency. These results prove the good physicochemical and mechanical stability and resistance of the Alu-wire for employing as the SPME fiber substrate.

In the following, the Ni/GO/NiTAM sorbent stability has been evaluated in various temperatures and media. The thermal stability of the Ni/GO/NiTAM has been studied by storing a few coated fibers at different temperatures, ranging from 0 to 60 °C for 2 h. Additionally, the chemical stability, adhesion, and swelling behavior of the Ni/GO/NiTAM sorbent have been examined by dipping a few coated fibers in acetone, deionized water, ethanol, methanol, NaCl, n-hexane, and THF for 4 h. Except for NaCl, no noticeable swelling and stripping of the sorbent have been detected in other cases. Therefore, the addition of salt damages the fiber sorbent and thus sharply reduces the fiber efficiency. These studies have been confirmed that the offered sorbent keeps its extraction capacity in many harsh conditions.

As mentioned, poor adhesion and pouring of the coating materials are some of the main problems of conventional SPME fibers. As it has been completely explained, the Ni/GO/NiTAM sorbent has shown good stability in different temperature and environmental conditions. It also did not strip off over time or during the sample solution stirring, even after several applications. In addition, the sorbent did not remove from the surface due to possible contact with hands or various objects or hits. According to the information obtained in section ‘3.1’, the formation of chemical bonds via the interaction between the functional groups of the coating and Alu-wire and also porosities created by the etching process creates the strong adhesion between the sorbent and the metal substrate.

### Comparison of the presented method efficiency with the other PAHs determination techniques

Great rigidity, fabrication simplicity, long durability, reusability, eco-friendliness, high effectiveness, high extraction capacity and good recovery are the main advantages of the suggested fiber compared to the commercial ones. The fabrication cost of each fiber is less than 35 €, which is very affordable compared to the commercial fibers, which have an average price of about 175 €. Accordingly, due to the reusability of the Alu-Ni/GO/NiTAM fiber, the cost of each analysis will reduce. The comparison results of the proposed procedure with some of the other similar and non-similar PAHs extraction and determination methods, at optimum conditions, are summarized in Table 3. Wide linear range, low values of LODs and RSDs, high extraction efficiency in the complex matrices, and good recovery was achieved utilizing the designed Alu-Ni/GO/NiTAM-SPME fiber. The extraction time was also shorter than the similar methods. In addition, the comparison of the results with the widely used SPME fibers also proves the better efficiency of the Alu-Ni/GO/NiTAM-SPME fiber. Besides, generally, the SPME technique has less solvent consumption than the D-µ-SPE method, and also its devices are much cheaper than the stir bar sorptive extraction (SBSE) method. Therefore, the proposed procedure can be comfortably used for the simultaneous and rapid analysis of the selected analytes in complex matrices.

**Table 3:** Comparison of the method extraction efficiency with the other PAHs determination techniques

Analytical technique	Fiber coating	Samples	LR (µg L^–1^)	LOD (µg L^–1^)	RSD%	Extraction time (min)	Refs.
DI-SPME-GC-FID	DVB/CAR/PDMS	Lake water	0.003‒0.10	0.003‒0.030	0.30‒19.00	100	[52]
DI-HS-GC–MS	PDMS/DVB	Water	0.30‒10.00	0.07‒0.32	0.60‒24.29	80	[53]
HS-GC-FID	PDMS/DVB	Aqueous sample	0.10‒200.00	0.03‒1.00	5.00‒13.00	30	[54]
D-μ-SPE- GC‒MS	Zeolitic material	Water	2.08–208.00	0.10–0.89	0.03–6.49	10	[55]
HS-SPME-HPLC‒UV	3-(trimethoxysilyl)-1-propanthiol	Water	1.00‒500	0.03–1.00	4.10–6.70	20	[56]
DI-SPME-HPLC-FLD	PDMS/DVB	Water	0.40‒169.9	0.07‒0.99	9.90–13.10	90	[57]
DI-SPME-HPLC–UV	CFYM	Groundwater; River water	0.005–100.00	0.14‒1.83	4.40‒8.20	90	[58]
DI-coiled-SPME-HPLC–UV	Bone wastes	Tab water; Well water; Roadside soil	0.01–99.00	0.03–0.10	3.10‒9.00	70	[59]
DI-SPME-HPLC‒UV	Nickel/graphene oxide/nickel-complex	Tomato Potato Bread	0.10‒200.0	0.03–0.30	0.32‒2.94	30	This work

## Conclusion

In this study, an electroless nickelized aluchrom surface covered with a new coating of graphene oxide/nickel tetraazamacrocyclic complex has been introduced. Alu wires are abundantly available at affordable prices, and are much more economical than the commercial fused silica fibers and also do not have their non-stability and fragility problem. Besides, the introduced sorbent has high adhesion to the fiber surface (without using any glue, epoxy, or nafion) and thus will not possess the problem of coating stripping. Furthermore, the coating possessed a large surface area due to its porous structure and demonstrated high adsorption capacity for the selected PAHs. Simple construction, high durability, easy handling, high rigidity, nonspecific storage condition, eco-friendliness, and high precision are some of the other main properties of the suggested fiber. The presented technique also illustrates wide linear ranges, good intra-day and inter-day precision, low detection limits, and high recoveries (more than 83%) for the PAHs analysis. Given the results, the method's capability for the simultaneous measurement of the target species in different matrices has been proved. Therefore utilizing the method for the routine analysis of PAHs is suggested.

## Supplementary Information

**Additional file 1: Fig. S1.** The optimized parameters during the fiber fabrication process: kind of fiber coating (A), etching reagent (B), etching time (C), concentration of NiCl_2_ (D), pH of plating solution (E), electroless nickel coating time (F), GO dispersion (G), pH of GO dispersion (H), GO coating time (I), stirring rate of GO dispersion (J).

**Additional file 2: Fig. S2.** The effect of important experimental factors on the proposed method efficiency attained by utilizing Alu-Ni/GO/NiTAM-SPME fiber: extraction time (A), desorption solvent (B), desorption solvent volume (C), desorption time (D), pH of sample solution (E) and stirring rate of sample solution (F).

**Additional file 3: Fig. S3.** Evaluation of carry-over effect at the optimized conditions.

**Additional file 4: Fig. S4.** Chromatograms obtained with the proposed Alu-Ni/GO/NiTAM-SPME fiber for the tandoori Sangak bread sample (I) and the same sample spiked with 25.0 µg L^–1^ of the selected PAHs (II).

## Data Availability

Adequate and clear descriptions of the applied materials and tools are provided in the materials and method section of manuscript. In addition, the obtained data is clearly justified by mentioning the figures and tables in the manuscript.

## Abbreviations

Aluchrom: Alu
BET: Brunauer Emmett teller
DI-SPME: Direct immersion solid phase microextraction
EDX: Energy Dispersive X-ray
EF: Enrichment factor
FE-SEM: Field emission scanning electron microscopes
FT-IR: Fourier transform infrared spectroscopy
HPLC: High-performance liquid chromatography
LOD: Limit of detection
LOQ: Limit of quantification
Ni/GO/NiTAM: Nickel/graphene oxide/nickel tetraazamacrocyclic
RSD: Relative standard deviation
SPME: Solid-phase microextraction
PAHs: Polycyclic aromatic hydrocarbons
XRD: X-ray diffraction

## References

[1] Wang C, Zhou S, Song J, Wu S (2020). Human health risks of polycyclic aromatic hydrocarbons in the urban soils of Nanjing, China. Sci Total Environ.

[2] Behzadi M (2021). Facile fabrication and application of poly(ortho-phenetidine) nanocomposite coating for solid-phase microextraction of carcinogenic polycyclic aromatic hydrocarbons from wastewaters. Ecotoxicol Environ Saf..

[3] Li C, Li C, Yu H, Cheng Y, Xie Y, Yao W, Yahui G, Qian H (2020). Chemical food contaminants during food processing: sources and control. Crit Rev Food Sci Nutr..

[4] Zelinkova Z (2015). The occurrence of 16 EPA PAHs in food—a review. Polycycl Aromat Comp.

[5] Singh L, Agarwal T, Gandara JS (2020). PAHs, diet and cancer prevention: cooking process driven-strategies. Trends Food Sci Technol.

[6] Berk Z. Food process engineering and technology. Chapter 24: Frying, baking, roasting. Elsevier. 2009. P. 525‒531.

[7] Paris A, Ledauphin J, Poinot P, Gaillard JL (2018). Polycyclic aromatic hydrocarbons in fruits and vegetables: origin, analysis and occurrence. Environ Pollut.

[8] Abdel-Shafy HI, Mansour MSM (2016). A review on polycyclic aromatic hydrocarbons: source, environmental impact, effect on human health and remediation. Egypt J Pet.

[9] Razmi H, Khosrowshahi EM, Farrokhzadeh S (2017). Introduction of coiled solid phase microextraction fiber coated by mesoporoussilica/cetyltrimethylammoniumbromide for ultra-trace environmental analysis. J Chromatogr A.

[10] Sun X, Tan J, Ding H, Tan X, Xing J, Xing L, Li Z (2018). Detection of polycyclic aromatic hydrocarbons in water samples by annular platform-supported ionic liquid-based headspace liquid-phase microextraction. J Anal Methods Chem.

[11] Kamal AE, Shimizu K (2021). Determination of polycyclic aromatic hydrocarbons (PAHs) in smoking cessation aids by using high-performance liquid chromatography. Anal Biochem..

[12] Singhad A, Jhabc RR, Kamala R, Kesavachandrana C, Patelb DK (2021). Dispersive liquid–liquid microextraction for the analysis of specific marker compounds in human exposed with polyaromatic hydrocarbons (PAHs). Microchem J..

[13] Jalili V, Barkhordari A, Ghiasvand A (2020). A comprehensive look at solid-phase microextraction technique: a review of reviews. Microchem J..

[14] Huang S, Chen G, Ye N, Kou X, Zhu F, Shen J, Ouyang G (2019). Solid-phase microextraction: an appealing alternative for the determination of endogenous substances—a review. Anal Chim Acta.

[15] Al-Khshemawee H, Du X, Agarwal M, Yang JO, Ren YL (2018). Application of direct immersion solid-phase microextraction (DI-SPME) for understanding biological changes of Mediterranean fruit fly (Ceratitiscapitata) during mating procedures. Molecules.

[16] Jagirani MS, Soylak M (2020). A review: recent advances in solid phase microextraction of toxic pollutants using nanotechnology scenario. Microchem J..

[17] Patinha DJS, Silvestre AJD, Marrucho IM (2019). Poly(ionic liquids) in solid phase microextraction: recent advances and perspectives. Prog Polym Sci..

[18] Arshadi M, Shakeri H, Salvacion JWL (2016). A green adsorbent for the removal of BTEX from aqueous media. RSC Adv.

[19] Dil EA, Ghaedi M, Mehrabi F, Tayebi L (2021). Highly selective magnetic dual template molecularly imprinted polymer for simultaneous enrichment of sulfadiazine and sulfathiazole from milk samples based on syringe–to–syringe magnetic solid–phase microextraction. Talanta.

[20] Yu C, Wu F, Luo X, Zhang J (2021). Porphyrin-based covalent organic framework coated stainless steel fiber for solid-phase microextraction of polycyclic aromatic hydrocarbons in water and soil samples. Microchem J..

[21] Hosseinzadeh R, Tahmasebi R, Farhadi K, Moosavi AAM, Jouyban A, Moosavi BM, Jouyban A, Badraghi J (2021). Novel cationic surfactant ion pair based solid phase microextraction fiber for nano-level analysis of BTEX. J Colloids Surf B.

[22] Du J, Zhang R, Wang F, Wang X, Du X (2020). Template-directed fabrication of zeolitic imidazolate framework-67 derived coating materials on nickel/titanium alloy fiber substrate for selective solid-phase microextraction. J Chromatogr A.

[23] Razmi H, Pasandideh Y (2020). Introduction of commercial heating elements of resistance metal alloys as the novel solid-phase microextraction fibers for chromatographic monitoring of organic pollutants. J Iran Chem.

[24] Pasandideh Y, Razmi H (2020). Introduction of a biowaste/graphene oxide nanocomposite as a coating for a metal alloy based SPME fiber: application to screening of polycyclic aromatic hydrocarbons. Arab J Chem.

[25] Zhang X, Ma X, Li X, Li C, Wang R, Chen M (2018). Development of ultra-sensitive method for determination of trace atrazine herbicide in environmental water using magnetic graphene oxide-based solid-phase extraction coupled with dispersive liquid-liquid microextraction prior to gas chromatography-mass spectrometry. Water Air Soil Pollut.

[26] Merkle S, Kleeberg K, Fritsche J (2015). Recent developments and applications of solid phase microextraction (SPME) in food and environmental analysis—a review. Chromatography.

[27] Mo Z, Pang Y, Yu L, Shen X (2021). Membrane-protected covalent organic framework fiber for direct immersion solid-phase microextraction of 17beta-estradiol in milk. Food Chem..

[28] Serpa AG, Fernandez IP, Pasan J, Pino V (2019). Metal organic frameworks as key materials for solid-phase microextraction devices—a review. Separations.

[29] Musarurwa H, Tavengwa NT (2021). Extraction and electrochemical sensing of pesticides in food and environmental samples by use of polydopamine-based materials. Chemosphere.

[30] Pournaghi-Azar MH, Razmi HN (1998). Voltammetricbehaviour and electrocatalytic activity of the aluminum electrode modified with nickel and nickel hexacyanoferrate films, prepared by electroless deposition. J Electroanal Chem.

[31] Hummers WS, Offeman RE (1958). Preparation of graphitic oxide. J Am Chem Soc.

[32] Basiuk VA, Alzate CN, Henao HLV, Rybak AEV, Basiuk EV (2016). Coordination functionalization of graphene oxide with tetraazamacrocyclic complexes of nickel(II): generation of paramagnetic centers. Appl Surf Sci.

[33] Guarino C, Zuzolo D, Marziano M, Conte B, Baiamonte G, Morra L, Benotti D, Gresia D, Stacul ER, Cicchella D, Sciarrillo R (2019). Investigation and assessment for an effective approach to the reclamation of polycyclic aromatic hydrocarbon (PAHs) contaminated site: SIN Bagnoli, Italy. Sci Rep.

[34] Kamalabadi M, Kamankesh M, Mohammadi A, Hadian Z, Ferdowsi R (2019). Contamination and daily intake of polycyclic aromatic hydrocarbons in Iranian bread samples. Polycycl Aromat Comp.

[35] Matadha NY, Mohapatra S, Siddamallaiah L, Udupi V, Gadigeppa S, Raja DP (2019). Uptake and distribution of fluopyram and tebuconazole residues in tomato and bell pepper plant tissues. Environ Sci Pollut Res.

[36] Tuteja G, Rout C, Bishnoi NR (2011). Quantification of polycyclic aromatic hydrocarbons in leafy and underground vegetables: a case study around Panipat city, Haryana. India Environ Sci Technol.

[37] Moradi V, Seyedain SMA, Shakoori A, Hoseyni SE (2020). Development of a GC-MS method for determination of various polycyclic aromatic hydrocarbons in Iranian traditional and semi-industrial Taftoon bread. J Hazard Mater.

[38] ICH harmonized tripartite guideline, Validation of analytical procedure: text and methodology Q2(R1), Parent Guideline dated 27 October 1994, Complementary Guideline on Methodology dated 6 November 1996 incorporated in November 2005. https://www.gmp-compliance.org/files/guidemgr/Q2(R1).pdf.

[39] Lavanya J, Gomathi N (2016). Synthesis and characterization of nickel oxide/graphene sheet/graphene ribbon composite. AIP Conf Proc.

[40] Choi E, Kim J, Cui Y, Choi K, Gao Y, Han S, Pyo SG, Yoon S (2017). Effect of the graphene oxide reduction temperature on supercapacitor performance. Electron Mater Lett..

[41] Karabork M, Zubair RM, Urus S, Tumer M (2018). Synthesis and characterization of graphene oxide-based hybrid ligand and its metal complexes: Highly efficient sensor and catalytic properties. Appl Organomet Chem.

[42] What is Etching? Black Church Print Studio. 2020; https://www.blackchurchprint.ie/media/130330779334_What%20is%20Etching.pdf.

[43] Xu HL, Li Y, Jiang DQ, Yan XP (2009). Hydrofluoric acid etched stainless steel wire for solid-phase microextraction. Anal Chem.

[44] MM5017: Electronic materials, devices and fabrication, Lecture 26: Etching and deposition. 2020; https://staff-old.najah.edu/sites/default/files/Lec26.pdf.

[45] Nguemte PM, Noumsi IMK, Wafo GVD, Djocgoue PF, Wanko A (2020). Modelling PAHs transfer from polluted soil to herbaceous species in phytoremediation attempts. Water.

[46] Konios D, Stylianakis MM, Stratakis E, Kymakis E (2014). Dispersion behaviour of graphene oxide and reduced graphene oxide. J Colloid Interface Sci.

[47] Zheng X, Zhao Y, Wenwu, Zheng H, Gao L. Application of graphene and its compounds in pretreatment of environmental samples. IOP Conference Series Earth Environ Sci. 2021;687:012064.

[48] Khorrami AR, Pasandideh Y (2016). Preparation of a novel sol-gel molecularly imprinted polymer with dummy template for online solid-phase extraction of patulin from apple juice samples. Int J Anal Tech.

[49] Kremser A, Jochmann MA, Schmidt TC (2015). PAL SPME Arrow-evaluation of a novel solid-phase microextraction device for freely dissolved PAHs in water. Anal Bioanal Chem.

[50] Calero AM, Ayala JH, Gonzalez V, Afonso AM (2008). Ionic liquids as desorption solvents and memory effect suppressors in heterocyclic aromatic amines determination by SPME–HPLC fluorescence. Anal Bioanal.

[51] Wang J, Chen Z, Chen B (2014). Adsorption of polycyclic aromatic hydrocarbons by graphene and graphene oxide nanosheets. Environ Sci Technol.

[52] Suterio N, do Carmo S, Budziak D, Merib J, Carasek E (2018). Use of a natural sorbent as alternative solid-phase microextraction coating for the determination of polycyclic aromatic hydrocarbons in water samples by gas chromatography-mass spectrometry. J Braz Chem Soc.

[53] Bianchin JN, Nardini G, Merib J, Dias AN, Martendal E, Carasek E (2012). Simultaneous determination of polycyclic aromatic hydrocarbons and benzene, toluene, ethylbenzene and xylene in water samples using a new sampling strategy combining different extraction modes and temperatures in a single extraction solid-phase microextraction-gas chromatography–mass spectrometry procedure. J Chromatogr A.

[54] Wei M, Jen JF (2007). Determination of polycyclic aromatic hydrocarbons in aqueous samples by microwave assisted headspace solid-phase microextraction and gas chromatography/flame ionization detection. Talanta.

[55] Ciric S, Mitic V, Jovanovic S, Ilic M, Nikolic J, Stojanovic G, Jovanovic VS (2018). Dispersive micro-solid phase extraction of 16 priority polycyclic aromatic hydrocarbons from water by using thermally treated clinoptilolite, and their quantification by GC-MS. Microchim Acta.

[56] Zare F, Ghaedi M, Daneshfar A (2015). The headspace solid-phase microextraction of polycyclic aromatic hydrocarbons in environmental water samples using silica fiber modified by self-assembled gold nanoparticles. Anal Methods.

[57] Acevedo JJJ, Sayadi MNK, Polo Diez LM (2013). A new SPME thermal desorption interface for HPLC. J Anal Sci Meth Instrum.

[58] Razmi H, Farrokhzadeh S (2018). Facile preparation of a chicken feet yellow membrane coated fiber for application in solid-phase microextraction. Sep Sci Plus.

[59] Razmi H, Farrokhzadeh S (2017). Introduction of a coiled solid-phase microextraction fiber based on a coating of animal bone waste for chromatographic analysis. J Sep Sci.

