# Supplementary figures and images for: Preparation of a new coating of graphene oxide/nickel complex on a nickelized metal surface for direct immersion solid phase microextraction of some polycyclic aromatic hydrocarbons
Source: BMC Chem. 2021 Oct 16;15(1):56. doi: 10.1186/s13065-021-00783-w (PMC8520613; doi:10.1186/s13065-021-00783-w)

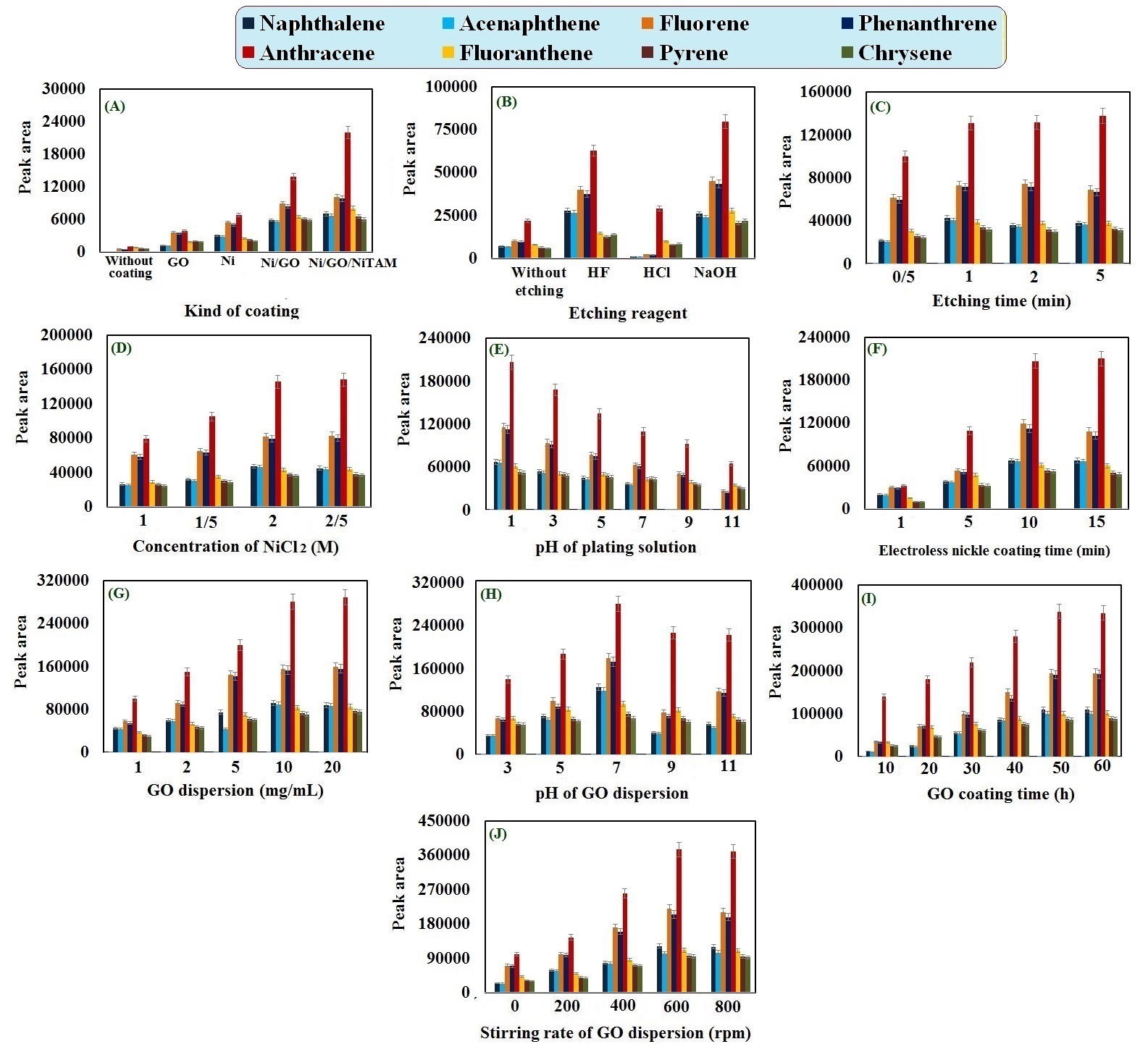

Supplement: Supplementary file 1 — Additional file 1: Fig. S1. The optimized parameters during the fiber fabrication process: kind of fiber coating (A), etching reagent (B), etching time (C), concentration of NiCl2 (D), pH of plating solution (E), electroless nickel coating time (F), GO dispersion (G), pH of GO dispersion (H), GO coating time (I), stirring rate of GO dispersion (J). [file 13065_2021_783_MOESM1_ESM.jpg]

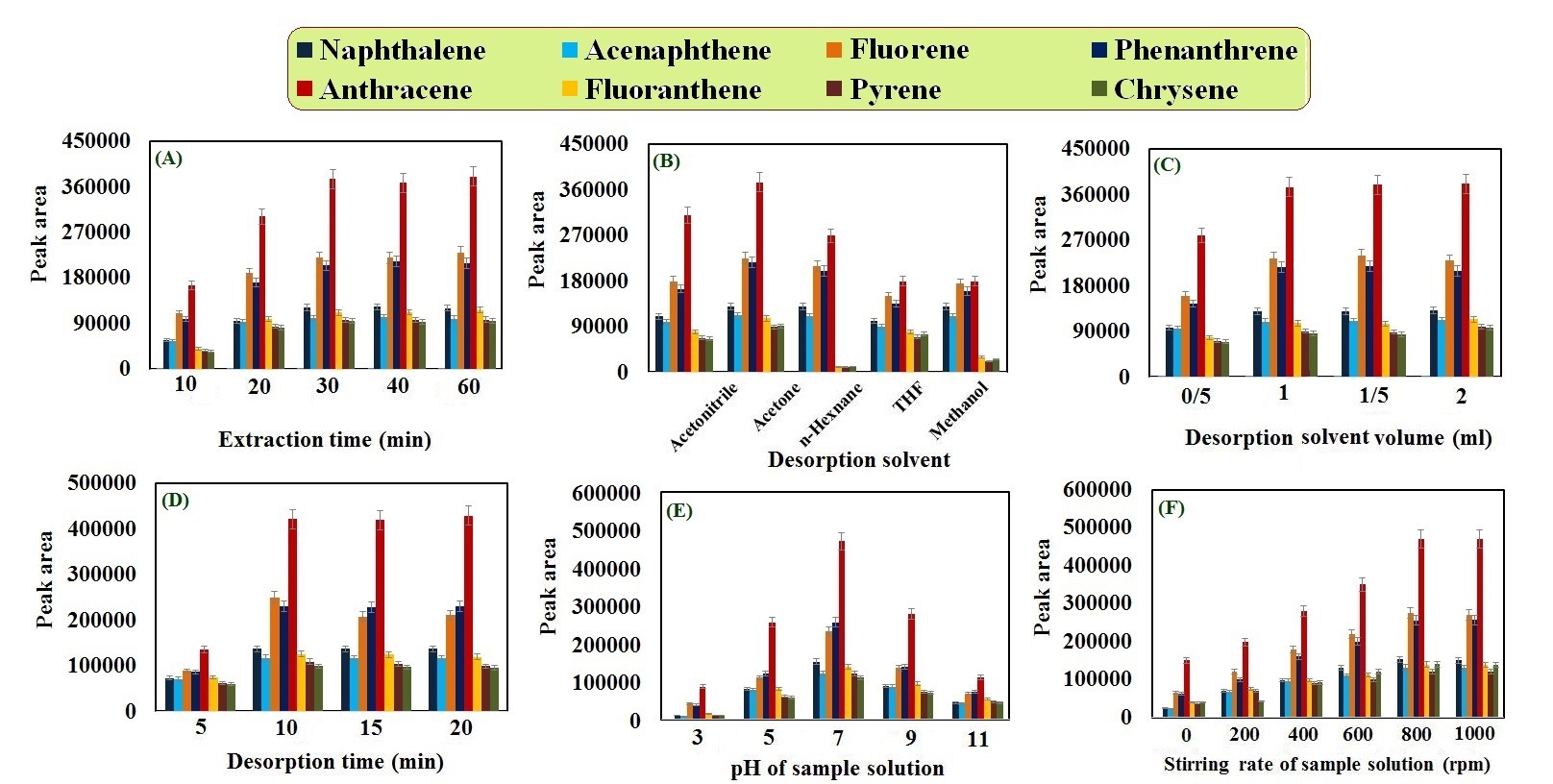

Supplement: Supplementary file 2 — Additional file 2: Fig. S2. The effect of important experimental factors on the proposed method efficiency attained by utilizing Alu-Ni/GO/NiTAM-SPME fiber: extraction time (A), desorption solvent (B), desorption solvent volume (C), desorption time (D), pH of sample solution (E) and stirring rate of sample solution (F). [file 13065_2021_783_MOESM2_ESM.jpg]

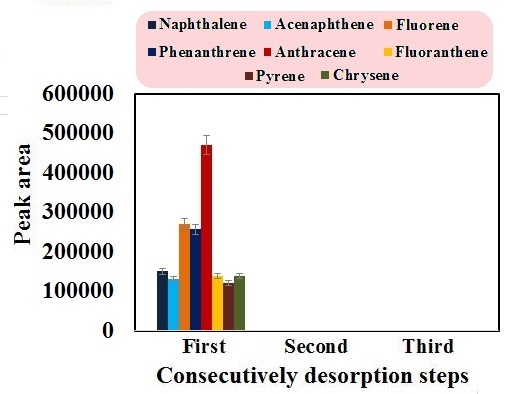

Supplement: Supplementary file 3 — Additional file 3: Fig. S3. Evaluation of carry-over effect at the optimized conditions. [file 13065_2021_783_MOESM3_ESM.jpg]

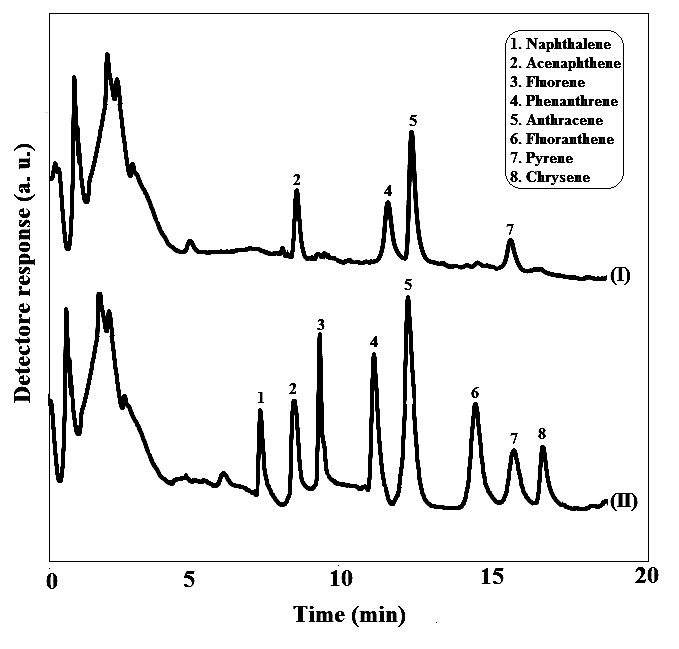

Supplement: Supplementary file 4 — Additional file 4: Fig. S4. Chromatograms obtained with the proposed Alu-Ni/GO/NiTAM-SPME fiber for the tandoori Sangak bread sample (I) and the same sample spiked with 25.0 µg L–1 of the selected PAHs (II). [file 13065_2021_783_MOESM4_ESM.jpg]
